# A Case-Case Comparison of *Campylobacter coli* and *Campylobacter jejuni* Infection: A Tool for Generating Hypotheses

**DOI:** 10.3201/eid0809.10.3201/eid0809.010187

**Published:** 2002-09

**Authors:** Iain A. Gillespie, Sarah J. O’Brien, Jennifer A. Frost, Goutam K. Adak, Peter Horby, Anthony V. Swan, Michael J. Painter, Keith R. Neal, the Campylobacter Sentinel Surveillance System Collaborators

**Affiliations:** * Public Health Laboratory Service (PHLS) Communicable Disease Surveillance Centre, London, United Kingdom; † PHLS Laboratory of Enteric Pathogens, London, United Kingdom; ‡ PHLS Statistics Unit, London, United Kingdom; § Manchester Health Authority, Manchester, United Kingdom; ¶ University of Nottingham, Nottingham, United Kingdom

**Keywords:** Campylobacter, epidemiology, surveillance, hypothesis generation, risk

## Abstract

Preventing campylobacteriosis depends on a thorough understanding of its epidemiology. We used case-case analysis to compare cases of *Campylobacter coli* infection with cases of *C. jejuni* infection, to generate hypotheses for infection from standardized, population-based sentinel surveillance information in England and Wales. Persons with *C. coli* infection were more likely to have drunk bottled water than were those with *C. jejuni* infection and, in general, were more likely to have eaten pâté. Important differences in exposures were identified for these two *Campylobacter* species. Exposures that are a risk for infection for both comparison groups might not be identified or might be underestimated by case-case analysis. Similarly, the magnitude or direction of population risk cannot be assessed accurately. Nevertheless, our findings suggest that case-control studies should be conducted at the species level.

Campylobacters are the most commonly reported bacterial cause of acute gastroenteritis in the industrialized world (1). In the United Kingdom (UK), laboratory reports of campylobacter have increased steadily since surveillance began in 1977; in 1999, >60,000 cases were reported (incidence rate 103.7 per 100,000). However, the true population burden of campylobacter infection is thought to be much higher. For every laboratory-confirmed case reported to national surveillance in England, an additional eight cases may be unrecognized (2). This estimate suggests that in 1999, approximately half a million people in the UK became ill with campylobacter enteritis. The cost to the nation of a case of campylobacter infection has been estimated as £314.00 (at 1994–95 prices) (3); in 1999 campylobacter infection probably cost the nation >£150 million (US$ 225 million). The clinical complications of campylobacter infection include toxic megacolon, hemolytic uremic syndrome, Reiter’s syndrome, and Guillain Barré syndrome, the most common cause of acute neuromuscular paralysis in the industrialized world (4).

 Although campylobacters were recognized as important pathogens >20 years ago, their epidemiology is still poorly understood (5–8). Eating poultry has long been a leading hypothesis for spread of campylobacter infection, but few case-control studies have identified it as a major risk factor except in a commercial context (9–11). An estimated 20% to 40% of sporadic disease might result from eating chicken (12,13). Although a variety of food vehicles and other risk factors have been reported in several case-control studies, most cases in these studies remain unexplained by the risk factors identified (5–11).

A difficulty, until recently, has been the lack of routine microbiologic characterization of clinical strains (14), which has militated against systematic study of the epidemiology of the different species and subtypes of campylobacter. Control and prevention strategies cannot be developed and implemented without proper understanding of the epidemiology of campylobacter infection. On May 1, 2000, an active, population-based sentinel surveillance scheme for campylobacter infections was initiated in England and Wales (15). Its aim is to generate hypotheses for human campylobacter infection by using a systematic, integrated epidemiologic and microbiologic approach. Twenty-two district health authorities are collaborating in the scheme, working with their hospital microbiology and local environmental health departments (Figure 1). The sentinel system covers a population of approximately 12.5 million and captures standardized information on approximately 15% of all laboratory-confirmed campylobacter infections in England and Wales. The health authorities are broadly representative of England and Wales as a whole.

**Figure 1:**
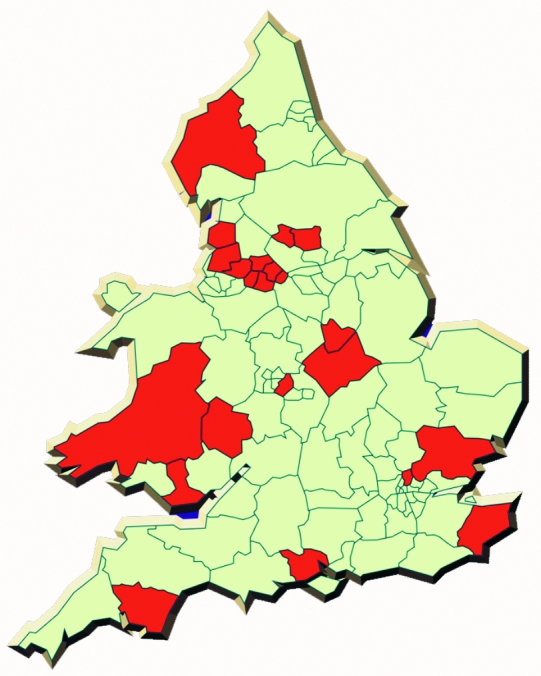
The health authorities in England and Wales participating in the sentinel surveillance scheme for Campylobacter.

 We have used case-case comparisons, an adaptation of conventional case-control methods, as suggested by McCarthy and Giesecke (16), to generate hypotheses concerning risk factors for campylobacter infection. We report results from the first year of the study and discuss the strengths and weaknesses of case-case analysis.

## Methods

Campylobacters isolated by National Health Service and Public Health Laboratory Service (PHLS) laboratories within the catchment area were referred to the Campylobacter Reference Unit of the PHLS Laboratory of Enteric Pathogens for speciation, serotyping, phage typing, and antibiotic resistance testing (17–20). A standard, structured clinical and exposure questionnaire was administered to each patient by the health or local authority as part of the routine investigation of foodborne infection. The questionnaire, which can be completed by the patient, captured demographic and clinical data, as well as travel history (foreign and domestic), food history (>20 exposures), milk (3 exposures) and water (8 exposures) consumption, recreational water activity, animal contacts, and other illness (either in the household or the community) during the 2 weeks before the onset of illness. Epidemiologic exposure data and microbiologic typing information were then collated centrally by the Gastrointestinal Diseases Division of the PHLS Communicable Disease Surveillance Centre.

The combined epidemiologic and microbiologic dataset, generated through the sentinel scheme, was analyzed by Stata version seven (Stata Corporation, College Station, TX). For the case-case analysis, illness in patients infected with *C. coli* was designated a “case;” patients infected with *C. jejuni* were designated as controls. Differences in demographic and clinical data were assessed by using Pearson’s chi-square test and the Student t test. Cases were excluded from analysis if a patient was infected with more than one campylobacter subtype (133 cases) or was confirmed as infected with *C. lari* (two patients) or *C. fetus* (one patient).

The date of onset of illness for cases was used to define the month of onset and approximations of the four seasons (spring, March–May; summer, June–August; autumn, September–November; winter, December–February) were calculated. Socioeconomic group, based on occupation, was determined by standard occupational classification (21). Additional categories were generated for persons who described their occupation as unemployed, preschool child, school child, student, homemaker, retired, or part time, and for those who were unable to work because of disabilities or long-term illness. Food exposures were coded to compare those who had eaten a particular food in the 2 weeks before onset of illness (once or more than once) with those who had not. Daily water consumption was coded to differentiate no exposure from 1–4, 5–9, and >10 glasses of water drunk. Patient age was classified in 10-year age groups. Persons with missing data were omitted from the analyses using those data.

Initially, comparisons between *C. coli* and *C. jejuni* cases were performed by single-risk variable analyses. Mantel-Haenszel odds ratios (OR) were calculated for each explanatory variable. Logistic regression was applied to obtain maximum likelihood estimates of the effect of exposures on the species-specific outcome, while the data were controlled for potential confounders. Variables with a p value <0.1 from the single-risk variable analysis were included initially. Stepwise exclusion was used to simplify the model: variables were removed one at a time and tested for significance by the likelihood ratio (LR) test. Potential interactions (among the main effects included in the initial logistic regression model were age, sex, and season) were also examined by using the LR chi-square test.

## Results

**Figure 2:**
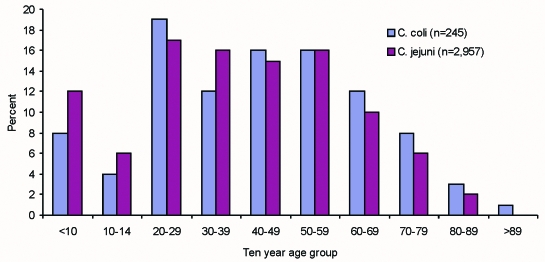
Age distribution of *Campylobacter coli* and *C. jejuni* cases reported to the sentinel surveillance scheme.

Epidemiologic data have been gathered for 7,360 laboratory-confirmed cases of campylobacter infection during the first year of the study (response rate 7,360 [76%] of 9,655). The median delay between onset of symptoms and completion of a questionnaire was 16 days. Case-patients ranged from <1 month to 99 years of age (Figure 2), and the overall sex distribution was even. Diarrhea (95%), abdominal pain (85%), and fever (78%) were the most commonly reported symptoms, with vomiting (35%) and bloody diarrhea (27%) reported less frequently. A total of 6,948 case-patients amassed 79,090 days of illness (mean 11), and 10% were hospitalized for an average of 5 days (range 1–42 days). Six hundred fifty-nine patients accumulated 3,048 hospital days. Five thousand one hundred seven patients reported absence from work or an inability to undertake normal activities for a total of 38,769 days (mean 8 days).

 Linked epidemiologic and microbiologic data are available for 3,764 cases. *C. jejuni* accounted for 3,489 (93%) of the cases, with 272 *C. coli* (7%), 2 *C. lari* (<1%), and 1 *C. fetus* (<1%) also reported. Case-patients with *C. coli* and *C. jejuni* infection did not differ with regard to sex, clinical symptoms, or duration of illness (Table 1). However, case-patients infected with *C. coli* tended to be older (mean 42.9 years) than patients with *C. jejuni* (mean 38.5 years) (p=0.001).

**Table 1:** Demographics, clinical symptoms, and severity of infections with *Campylobacter coli* and *C. jejuni*

Variable		Campylobacter species (%)		χ^2^	p value
		*C. coli* (n=272)	*C. jejuni* (n=3,489)		
Mean age		42.9	38.5	-	0.001
Male		123 (45)	1,734 (50)	2.02	0.16
Female		149 (55)	1,755 (50)		
Mean length of illness		11.4	11.3	-	0.92
Diarrhea	Yes	253 (96)	3,355 (98)	3.11	0.08
	No	10 (4)	73 (2)		
Bloody stools	Yes	73 (35)	964 (34)	0.07	0.79
	No	134 (65)	1843 (66)		
Vomiting	Yes	87 (37)	1249 (40)	1.00	0.32
	No	151 (63)	1885 (60)		
Abdominal pain	Yes	236 (93)	3,013 (92)	0.13	0.72
	No	19 (7)	265 (8)		
Fever	Yes	206 (84)	2,812 (86)	1.44	0.23
	No	40 (16)	440 (14)		
Seeking advice from a doctor	Yes	260 (97)	3,345 (98)	0.65	0.42
	No	8 (3)	76 (2)		
Hospitalized	Yes	23 (9)	358 (10)	0.97	0.32
	No	245 (91)	3,055 (90)		
Mean days off work/normal activities		6.7	7.6	-	0.05

Patients with *C. coli* infection were more likely to describe their ethnicity as Asian and to have traveled abroad in the 2 weeks before the onset of symptoms (single-risk variable analysis; Table 2). Patients with *C. coli* were also more likely to report having eaten specific types of meats (Halal meat [meat slaughtered according to Islamic law], meat pies, offal [organ meats], and pâté) and bottled water. They were less likely to have had contact with animals than were patients with *C. jejuni* infection. Persons with *C. coli* and those with *C. jejuni* infection did not differ with regard to eating chicken (89.8% vs. 90.8%; odds ratio [OR] 0.89; 95% confidence interval [CI] 0.58 to 1.36; chi square 0.59) or other types of poultry (23.6% vs. 19.7%; OR 1.26; 95% CI 0.91 to 1.74; chi square 0.16) in the 2 weeks before onset of illness.

**Table 2:** Risk exposures for *Campylobacter coli* infection, by single-risk variable analysis

Exposure	No. exposed (%)		Odds ratio	p value^a^	95% Confidence intervals
	*C. coli* (n=272)	*C. jejuni* (n=3,489)			
Summer	75 (27.6)	1,206 (34.6)	0.72	0.02	0.55 to 0.95
Dyfed Powys Health Authority	5 (1.8)	24 (0.70)	2.7	0.04	1.02 to 7.15
10-year age group (increasing)	-	-	1.10^b^	0.001^c^	1.04 to 1.17
Members of the armed forces	1 (0.37)	2 (0.06)	6.43	0.08	0.58 to 71.27
Retired persons	61 (22.4)	580 (16.6)	1.45	0.01	1.07 to 1.95
Preschool-aged children	14 (5.2)	288 (8.3)	0.60	0.07	0.35 to 1.05
Homemakers	16 (5.9)	131 (3.8)	1.60	0.08	0.94 to 2.73
South Asian ethnicity	21 (9.1)	168 (5.8)	1.63	0.04	1.01 to 2.61
European ethnicity	4 (1.7)	118 (4.1)	0.42	0.08	0.15 to 1.14
Travel abroad	76 (28.3)	653 (19.0)	1.68	0.0002	1.27 to 2.22
Halal meats	23 (10.7)	216 (7.3)	1.52	0.07	0.96 to 2.39
Meat pies	78 (33.9)	856 (27.9)	1.32	0.049	1.00 to 1.76
Offal (organ meat)	19 (8.7)	170 (5.6)	1.60	0.06	0.97 to 2.62
Pâté	42 (18.7)	397 (13.2)	1.51	0.02	1.06 to 2.14
Bottled water	150 (63.6)	1,646 (53.7)	1.51	0.003	1.14 to 1.98
Contact with animals	138 (51.7)	1,989 (57.8)	0.78	0.049	0.61 to 1.00

^a^Exposures where p<0.1 shown. ^b^Approximation to the odds ratio for a one-unit increase in 10-year age group. ^c^Derived from score test for trend of odds.

Patients with *C. coli* infection were more likely to have drunk bottled water than persons with *C. jejuni* infection and, in general, were more likely to have eaten pâté (logistic regression analysis; Table 3). Retired persons who ate meat pies were more likely to be infected with *C. coli* than *C. jejuni*, as were Asians who had traveled abroad in the 2 weeks before illness. Case-patients with *C. coli* infection were, in general, less likely to be ill in the summer, and men who traveled abroad in the 2 weeks before illness were more likely to be infected with *C. jejuni* infection.

**Table 3:** Independent risk exposures for *Campylobacter coli* infection: final logistic regression model^a^

Exposure	Odds ratio	p value	95% Confidence intervals
Summer	0.64	0.029	0.42 to 0.95
Summer (for participants 50–60 y of age)	3.10	0.013	1.27 to 7.59
South Asians who traveled abroad	9.70	0.006	1.89 to 49.73
Pâté	1.85	0.006	1.19 to 2.88
Pâté (for participants 50–60 y of age)	0.21	0.050	0.05 to 1.00
Meat pies eaten by retired persons	3.41	0.005	1.45 to 8.01
Bottled water	1.45	0.042	1.01 to 2.08
Men who traveled abroad	0.42	0.028	0.19 to 0.91
Male	1.05	0.804	0.72 to 1.53
Age (y)	1.00	0.586	0.99 to 1.02

^a^ Main effects not shown if p>0.05; data were controlled for a priori confounders of age and sex.

## Discussion

To our knowledge, this population-based sentinel surveillance system for campylobacter infection is unique because we have successfully linked detailed epidemiologic exposure information with detailed microbiologic strain characterization for a large sentinel population. Campylobacters are widely distributed in the environment, and this genus is adapted to a wide range of ecologic niches throughout the food chain (22). Microbiologic data show that the prevalence of different campylobacter species and subtypes varies between different potential sources of infection, including different animal species, foods, and water (23–27). Although *C. coli* infection accounts for a small proportion of laboratory-confirmed human campylobacter cases in England and Wales, the potential for prevention is substantial if the true population burden is much higher (3). Most case-control studies have so far sought to determine risk factors for sporadic infection with campylobacter and have not sought to differentiate between species (5–7). This distinction is important if *C. coli* and *C. jejuni* differ in their etiology or if the contribution of similar risk factors differs between the two species. If exposures are aggregated for different pathogenic campylobacter species, the contribution of risk factors unique to or predominantly associated with *C. coli* will be masked by the predominance of *C. jejuni* (in the study population: *C. jejuni*: *C. coli* approximately 10:1). This source of bias can be overcome by comparing the exposure characteristics of cases with *C. coli* infection with those of cases with *C. jejuni* infection. The data for cases with *C. jejuni* infection are then used to contrast with, rather than dilute, any observations for *C. coli* infection. Therefore, in generating hypotheses for infection, we identified potential species differences by adopting case-case analysis.

### Hypothesis: Bottled Water

 Case-patients with *C. coli* infection were more likely to report bottled water consumption than were those with *C. jejuni* infection. This observation is biologically plausible. Raw water can be contaminated with *C. coli* (28,29) and, while European legislation governing the marketing of natural mineral water makes it a condition that it be free from parasites and pathogenic organisms (30), testing for campylobacters is rarely undertaken (31). As the bottled water industry is large ($35 billion a year worldwide [32]) and expanding rapidly (consumption in the United States, which was 5 billion gallons in 2000, is predicted to increase to 7.3 billion gallons in 2005 [32]), an accurate assessment of the risk associated with these products is required. Our hypothesis-generating questionnaire did not distinguish between types of bottled water (e.g., spring or mineral, carbonated, or still), but these issues merit further investigation by case-control study.

### Hypothesis: Pâté

The finding that having eaten pâté was more likely to be reported by case-patients with *C. coli* infection than those with *C. jejuni* infection is also biologically plausible. Pork is often the main constituent of pâté, and *C. coli* is found in pigs (33). In a recent study of the occurrence of campylobacters in 400 freshly eviscerated porcine liver samples, 6% were infected with *Campylobacter* spp; most (67%) were *C. coli* (34). Pâté is a perishable comminuted meat product containing nitrite, and possibly nitrate, ascorbate, or both (35). While the use of such preservatives might deter the growth of spoilage microorganisms (assuming adequate storage conditions are maintained), vegetative pathogens might not be destroyed; therefore, the ultimate critical control point during production is likely to be effective heat treatment.

### Hypothesis: Meat Pies

The fact that retired people with *C. coli* infection were more likely to report having eaten meat pies is interesting. The types of meat in the pie fillings are not known, but the finding might point to the use of cheaper cuts of meat in these products.

### Hypothesis: Foreign Travel

 Persons from a South Asian ethnic background who had traveled abroad in the 2 weeks before onset of symptoms were more likely to have acquired a *C. coli* infection, but the reverse was true for men. This finding probably reflects the fact that travel abroad is simply a marker for activities or behavior while abroad, and a further study of the “travel cohort,” generated through the surveillance scheme, might provide a better indication of where the risks lie.

### Hypothesis: Seasonality

 Campylobacter infection has marked seasonality, and case-patients infected with *C. coli* were less likely to be ill in the summer than those infected with *C. jejuni*. As data accumulate, generating season-specific hypotheses might be possible, which may have implications for the time period over which analytic studies are performed.

### Sources of Bias

In interpreting the results from the sentinel surveillance system, likely sources of bias should be considered. Selection bias has been minimized by including all laboratory-confirmed cases of campylobacter infection identified by PHLS and National Health Service laboratories in the participating districts. Furthermore, both groups in the case-case comparison have been subjected to the same selection process, so selection bias should not influence our analysis.

The effect of time delays in reaching the patient, and hence recall bias for reported exposures, should be limited by close collaboration between the various participants in the scheme. While the time delay reported in this study introduces some recall bias, there is no reason to believe that recall is operating differently among patients infected with different species or among exposure groups, so that recall bias should not influence the case-case comparison.

### Interpreting Case-Case Analyses

A detailed account of the pros and cons of case-case analysis is provided by McCarthy and Giesecke (16), but two important points influence the interpretation of this type of study. The first is that exposures that are a risk for infection for both comparison groups will not be identified or might be underestimated. By using patients with campylobacter infection, albeit with a different species, as “controls,” we may obscure an association with the infection of interest because the controls might share some of the risk exposures with the cases. Thus, exposures common to both infections are controlled for by the study design.

The second is that traditionally controls are selected to provide an estimate of the exposure prevalence that would be seen in the cases if there were no association between the exposure and disease. Since our controls have been differentially selected by factors that are related to certain exposures, they might not be representative of the exposure prevalence of the population group from which the cases originated. We cannot, therefore, use comparisons between our cases and controls to make statements about the magnitude or direction of population risk.

## Conclusion

Our work has shown that important differences in exposures might exist for these two campylobacter species. This finding is not necessarily surprising. For example, nontyphoidal salmonellosis is well recognized to represent a large group of serotypes, each with its own distinctive epidemiology (36). Given this knowledge, conducting a case-control study with a case definition comprising *Salmonella* spp. is inconceivable. Why should the same not be true for *Campylobacter* spp.? The implications for analytic study design are that researchers should not aggregate different species, which may mask important species-specific risk factors. Thus, the comparison of two organisms thought to represent one disease with a common cause has provided new avenues for the epidemiologic investigation of human disease. Focused analytical studies, based on systematically generated hypotheses, determining etiologic fractions for the risk factors identified, will allow informed prevention strategies for human infection.
